# Invariant NKT Cells Act as an Adjuvant to Enhance Th2 Inflammatory Response in an OVA-Induced Mouse Model of Asthma

**DOI:** 10.1371/journal.pone.0119901

**Published:** 2015-04-01

**Authors:** Hanxiang Nie, Qiaoyu Yang, Guqin Zhang, Ailing Wang, Qing He, Min Liu, Ping Li, Jiong Yang, Yi Huang, Xuhong Ding, Hongying Yu, Suping Hu

**Affiliations:** 1 Department of Respiratory Medicine, Renmin Hospital of Wuhan University, Wuhan, China; 2 Department of Respiratory Medicine, Zhongnan Hospital of Wuhan University, Wuhan, China; 3 Wuhan University HOPE School of nursing, Wuhan, China; University of KwaZulu-Natal, SOUTH AFRICA

## Abstract

**Background:**

Invariant natural killer T cells (iNKT cells) are a unique subset of T lymphocytes and are considered to play an important role in the development of allergic bronchial asthma. Recently, iNKT cells were shown to play an immunoregulatory role in CD4^+^ and CD8^+^ T cell-mediated adaptive immune response. Allergen-specific Th2 inflammatory responses are an important part of the adaptive immune response in asthma. However, the regulatory functions of the Th2 inflammatory response in asthma have not been studied in detail.

**Method:**

In this study, we have investigated the regulatory functions of iNKT cells on the Th2 inflammatory response in an ovalbumin (OVA)-induced murine model of asthma.

**Results:**

Our results demonstrate that α-Galactosylceramide (α-GalCer) administration activated iNKT cells but could not induce the Th2 inflammatory response in wild-type (WT) mice. In the OVA-induced asthma model, α-GalCer administration and adoptive transfer of iNKT cells significantly augmented the Th2 inflammatory responses, including elevated inflammatory cell infiltration in the lung and bronchoalveolar lavage fluid (BALF); increased levels of IL-4, IL-5, and IL-13 in the BALF and splenocyte culture supernatant; and increased serum levels of OVA-specific IgE and IgG1. In addition, the Th2 inflammatory response was reduced, but not completely abrogated in CD1d^-/-^ mice immunized and challenged with OVA, compared with WT mice.

**Conclusion:**

These results suggest that iNKT cells may serve as an adjuvant to enhance Th2 inflammatory response in an OVA-induced murine model of asthma.

## Introduction

Asthma, a complex inflammatory disease of the airways, is traditionally driven by allergen-specific IgE and T helper (Th) 2 cells [1]. The allergen-specific Th2 cells orchestrate the inflammation process in asthma by producing Th2 cytokines, such as IL-4, IL-5, and IL-13, which enhance allergen-specific IgE synthesis, increase airway mucus production and the growth and differentiation of airway eosinophils, and directly induce the development of airway hyperresponsiveness (AHR), a cardinal feature of asthma [1]. However, this notion was challenged when the role for invariant natural killer T cells (iNKT cells) in the development of asthma was identified [2].

Invariant NKT cells constitute a unique subpopulation of T lymphocytes and express invariant T cell receptors (TCRs) that recognize glycolipid antigens (Ags) presented by CD1d, a non-polymorphic major histocompatibility complex (MHC) class I-like molecule [3]. Several studies have demonstrated the important roles of iNKT cells in the development of asthma. The percentage of iNKT cells is known to increase in the airways of asthmatics [4–6]. In the ovalbumin (OVA)-induced asthma model, the presence of iNKT cells is required for the development of allergen-induced AHR and airway inflammation [7, 8]. Recently, NKT cells have been shown to play an immunoregulatory role in the secondary phase of the adaptive immune response by mediating the production of cytokines and increase in the number of Ag-specific, conventional CD8^+^ T cells [9]. Fujii et al. [10] reported that activation of iNKT cells by α-Galactosylceramide (α-GalCer) rapidly stimulates complete maturation of dendritic cells (DCs) and that this stimulatory effect accounts for the induction of combined CD4^+^ Th1 and CD8^+^ T cell immunity to co-administered proteins. In addition, iNKT cells also play an important role in the establishment and regulation of CD4^+^ T cell-mediated adaptive immune responses [11–13]. Furthermore, allergen-specific Th2 inflammatory responses are an important part of the adaptive immune response in asthma [14] and our previous study showed that allergic airway inflammation was reduced but not completely abrogated when the activity of iNKT cells was inhibited in a mouse model of asthma [15]. Thus, we hypothesized that iNKT cells may not be essential but may play an immunoregulatory role in Th2 inflammatory responses in asthmatics.

To test this hypothesis, we have investigated Th2 inflammatory responses in the presence or absence of α-GalCer in wild-type (WT) mice without OVA immunization and challenge, as well as in OVA-induced asthma model. The Th2 inflammatory response was detected in CD1d^-/-^ and WT mice when immunized and challenged with OVA. Our results demonstrate that although α-GalCer administration can activate iNKT cells, it cannot induce the Th2 inflammatory response in WT mice without OVA immunization and challenge. On the other hand, the OVA-induced asthma model shows activation and increased number of iNKT cells and elevated cytokine production. Interestingly, α-GalCer administration and adoptive transfer of iNKT cells in this model markedly enhances the Th2 inflammatory responses, including elevated inflammatory cell infiltration in the lung and bronchoalveolar lavage fluid (BALF), increased levels of IL-4, IL-5, and IL-13 in the BALF and splenocyte culture supernatant, and increased serum levels of OVA-specific IgE and IgG1. Compared with the WT mice, CD1d^-/-^ mice showed a reduction but not complete ablation of the Th2 inflammatory response when immunized and challenged with OVA. Taken together, our results indicate that iNKT cells may serve as an adjuvant to enhance Th2 inflammatory responses in the OVA-induced mouse model of asthma.

## Materials and Methods

### Mice

WT female BALB/c mice (6–8 week old) were obtained from and maintained in the Animal Biosafety Level 3 Laboratory in the Center for Animal Experiment, Wuhan University (Wuhan, China). CD1d^-/-^ mice on BALB/c background were purchased from the Jackson Laboratory (Bar Harbor, ME). All mice were housed in environmentally controlled and specific pathogen-free conditions (22°C, 12 h light/12 h dark cycle). All animal care and handling protocols were approved by the Animal Welfare Committee of Wuhan University.

### OVA sensitization and airway challenge

WT BALB/c mice or CD1d^-/-^ mice were intraperitoneally sensitized on day 0 and 14 with 20 μg of chicken ovalbumin (OVA; grade V, Sigma, St Louis, MO, USA) emulsified in 2 mg of aluminum hydroxide (Thermo Scientific Pierce, Rockford, Rockford, IL, USA) in 200 μL of phosphate-buffered saline (PBS). Intranasal OVA challenges (100 μg/50 μL in PBS) were administered on days 25, 26, and 27. Age- and sex-matched control mice were similarly treated with PBS alone. The mice were sacrificed 24 h after the final challenge and their sera, BALF, lungs, and spleen were harvested and analyzed.

### α-GalCer treatment

A stock solution of α-GalCer (Enzo Life Sciences, Ann Arbor, MI) was diluted to 0.01 mg/mL in 0.5% polysorbate-20 and stored at -20°C. PBS or 2 μg α-GalCer was intraperitoneally administered to mice, 1 h before challenge on day 25.

### Adoptive transfer

The donor spleen mononuclear cells (MNCs) were harvested 24 h after the final challenge from the OVA-induced asthmatic mice. iNKT cells (3 × 10^6^), purified from spleen MNCs using PE-PBS57/CD1d tetramers (purity was about 76% by flow cytometry for PE-PBS57/CD1d tetramer-positive cells double stained with PeCy5-TCR-β), were adoptively transferred into BALB/c mice via tail vein injections, 1 h before challenge on day 25.

### Analysis of the cellular composition of BALF

BALF was collected from mice to analyze the cellular components and cytokine production in the lung. Briefly, mice were sacrificed 24 h after the final OVA challenge, and their tracheas were immediately lavaged three times via a catheter with 0.5 mL aliquots of PBS containing 0.6 mM EDTA. The BALF collected was centrifuged at 400 × *g* for 5 min at 4°C and the supernatant was stored at -80°C until the assay for cytokine levels. The recovered cells were counted and washed. Differential cell counts were assessed on cytological preparations obtained by cytocentrifugation (TXD3 cytocentrifuge, Xiangyi, Changsha, China) of 100 μL of the diluted BALF (1 × 10^6^ cells/mL in ice-cold saline-EDTA). Slides were stained with May-Grunwald Giemsa (Jiancheng, Nanjing, China). At least 400 cells were counted for each preparation. The number of eosinophils, macrophages, neutrophils, and lymphocytes was expressed as the absolute number from the total cell counts.

### Lung histopathology and goblet cell hyperplasia

The right lungs were removed after BAL and immediately fixed in 4% buffered paraformaldehyde for 2 d at room temperature. The specimens were then dehydrated and embedded in paraffin. Sections were then cut into 4-μm slices for routine hematoxylin-eosin (H&E) staining and periodic acid-Schiff (PAS) staining (Baso, Taiwan, China). Peribronchial and perivascular inflammation was assessed using light microscopy at 100× magnification. Goblet cell hyperplasia was determined by counting the number of PAS-positive cells in the epithelium of the central airway on a digital image at 200× magnification. Four sections were evaluated per lung. The circumference of the airway at the basement membrane was measured by National Institutes of Health (NIH) image. The results were expressed as the number of PAS-positive cells per unit length (mm) of the basement membrane.

### Cell isolation and culture

Mice were sacrificed 24 h after the final OVA challenge. Their lungs were removed, cut into small pieces, digested in collagenase I (1 mg/mL, Invitrogen, California, USA) at 37°C for 1 h, and filtered through a 100-μm nylon net filter. The lung MNCs were isolated by centrifugation at 800 × *g* for 20 min at room temperature in a Lymphoprep gradient (density = 1.081 mg/mL; TBD, Tianjin, China). Splenocytes were obtained by pressing the spleen through a 100 μm nylon net filter, and erythrocytes were lysed using an red blood cell lysis buffer (Sigma). The suspension of single spleen cells from immunized and control mice was cultured in duplicates at a density of 4 × 10^6^ cells/mL in RPMI 1640 medium supplemented with 10% fetal bovine serum (FBS; Life Technologies), 1 mM sodium pyruvate, 2 mM L-glutamine, 100 U/mL penicillin, and 100 mg/mL streptomycin (complete medium) or with OVA (500 μg/mL) in complete medium. After 72 h of incubation, cell culture supernatants were collected to evaluate IL-4, IL-5, and IL-13 production using ELISA kits (eBioscience, San Diego, CA).

### ELISA and immunoglobulin analysis

Quantification of IL-4, IL-5, and IL-13 in the BALF was accomplished by using a commercially available ELISA kit, according to the manufacturer’s instructions (eBioscience, San Diego, CA, USA). For immunoglobulin (Ig) analysis, the serum was harvested 24 h after the final OVA challenge to determine the levels of OVA-specific IgE and IgG1 by ELISA. Briefly, 96-well plates (Corning Costar, New York, NY, USA) were coated with 200 μg/mL of OVA diluted in 0.1 M NaHCO_3_ (pH 8.3). After 3 h of incubation at 37°C, the plates were washed 6 times and blocked with 10% FBS in PBS for 1 h at 37°C. Serum samples were then incubated overnight at 4°C. After 6 washes with PBS containing 0.05% Tween-20, biotinylated antibodies against mouse IgE or IgG1 (Biolegend, San Diego, CA, USA) were added to the respective wells. After incubation for 1 h at room temperature, the plates were washed 6 times and incubated with an avidin-peroxidase complex (Sigma) for 30 min at room temperature. After the final washes, tetramethylbenzidine (Sigma) was added and the color was allowed to develop for 15–20 min. The reaction was stopped by adding 2 M H_2_SO_4_ and the absorbance intensity was read at 450 nm using a microplate reader (Tecan, Clontarf, Australia). Serum levels of OVA-specific Igs are expressed as the optical density.

### Flow cytometric analysis

Lung MNCs were resuspended in the FACS buffer (1–2 × 10^6^ cells/mL). Cells were first blocked with anti-CD16/CD32 antibody (clone 2.4G2; BD Biosciences, San Diego, CA) and then labeled with isotype controls or the following antibodies: FITC-IL-4, FITC-IFN-γ, PeCy5-TCR-β (eBioscience), PE-PBS-57 (α-GalCer analog)/CD1d tetramer (NIH tetramer core facility). Dead cells were excluded by forward scatter and side scatter. iNKT cells were measured as PBS-57/CD1d tetramer and TCR-β double-positive cells. For intracellular cytokine staining, lung MNCs were cultured for 4 to 6 h with 500 ng/mL ionomycin and 50 ng/mL phorbol 12-myristate 13-acetate (PMA) in the presence of monension (1 μL/mL). Cells were harvested and washed, and intracellular staining was performed according to the manufacturer’s protocol (eBioscience). Cells were analyzed by flow cytometry (Epics Altra; Beckman, Seattle, WA).

### Statistical analyses

All data are expressed as mean ± SD. Statistical significance between two groups was determined using student’s unpaired *t*-test. Data were analyzed using the GraphPad Prism 5 (GraphPad Software Inc, San Diego, CA, USA) software. A *P* < 0.05 was considered statistically significant. A “*” denotes *P* < 0.05 and “**” denotes *P* < 0.01. Presented data are representative of at least 3 separate and repeated experiments.

## Results

### Activation of iNKT cells by α-GalCer cannot induce Th2 inflammatory responses in WT mice without OVA immunization

**Fig 1:**
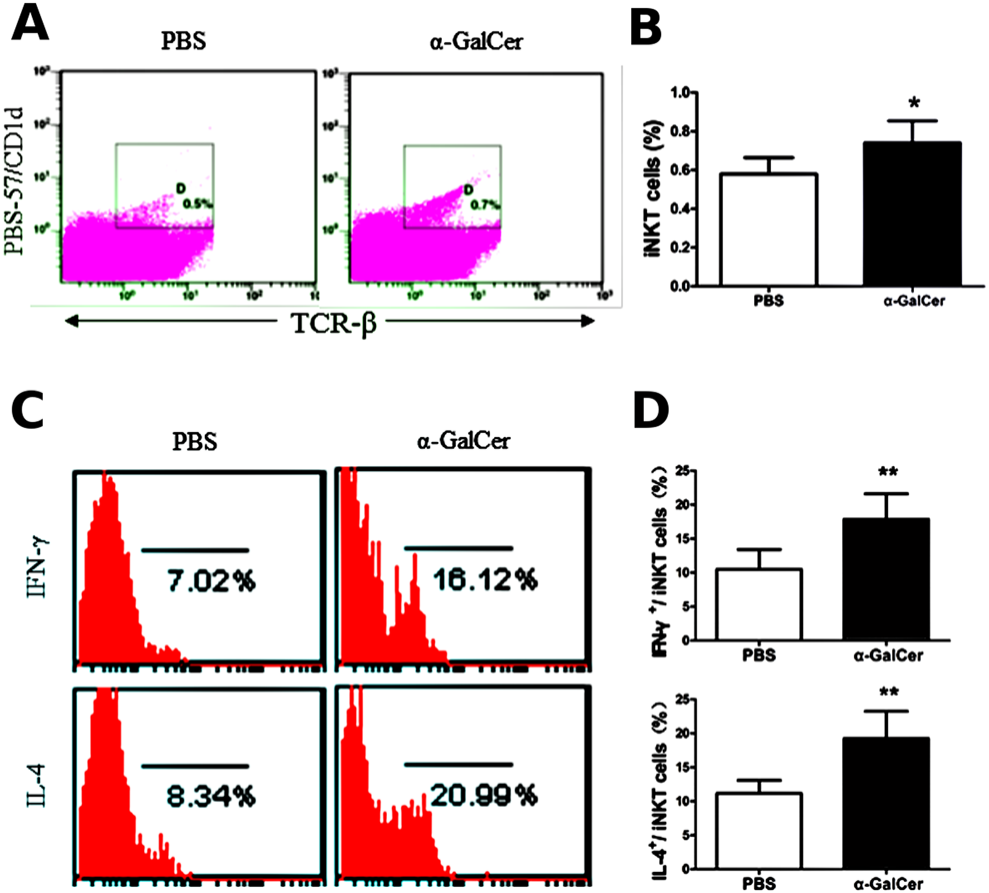
α-GalCer administration activates iNKT cells in wild-type (WT) mice. **A**. Lung iNKT cells were confirmed by PBS/CD1d tetramers and TCR-β staining in WT mice treated with α-GalCer or PBS using flow cytometry. The gating used for iNKT cells (PBS-57/CD1d tetramers^+^ TCR-β^+^) (gate D) and the corresponding percentages are shown in each dot plot. **B**. Percentage of iNKT cells in lung MNCs from WT mice treated with α-GalCer or PBS. N = 5 per group and *P < 0.05. **C**. Lung iNKT cells producing IFN-γ and IL-4. PBS-57/CD1d and TCR-β double-positive cells were examined for IFN-γ (top) and IL-4 (bottom) secretion. The numbers below the parentheses indicate the percentage of positive cells. **D**. Percentage of IFN-γ- and IL-4-expressing iNKT cells in the lung. N = 5 per group and **P < 0.01.

**Fig 2:**
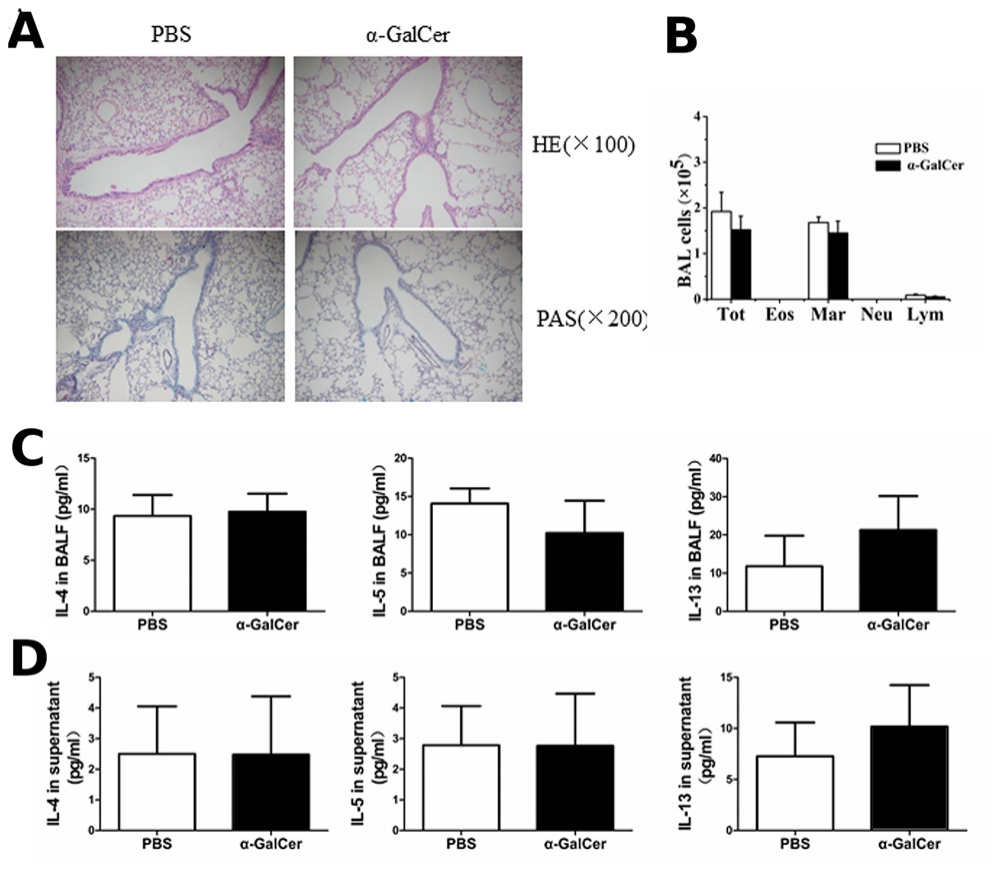
α-GalCer administration cannot induce the Th2 inflammatory response in WT mice. A. Histopathological analysis using the hematoxylin-eosin (H&E) staining and periodic acid-Schiff (PAS) staining of lung tissue sections from mice treated with α-GalCer or PBS. **B**. Total cell and differential cell counts in the BALF were obtained using a standard hemocytometer. N = 5 per group. Tot, total cell counts; Mar, macrophages; Eos, eosinophils, Neu, neutrophils; and Lym, lymphocytes. **C**. BALF were collected 3 days after α-GalCer or PBS administration and cytokine (IL-4, IL-5, and IL-13) production was analyzed by ELISA. N = 4–5 per group. **D**. Splenocytes were obtained from mice with α-GalCer or PBS administration and re-stimulated with 500 μg OVA *in vitro*. After 72 h, culture supernatants were collected and cytokine production was analyzed by ELISA. N = 5 per group.

α-GalCer is a specific ligand of iNKT cells, and is often used to investigate the role of iNKT cells in the immune responses involved in tumors, autoimmunity, and asthma [15–17]. To determine whether iNKT cells alone can induce the Th2 inflammatory response in WT mice in the absence of the peptide antigen, 2 μg of α-GalCer or PBS was administered intraperitoneally without OVA sensitization. Three days later, the number and activity of iNKT cells in the lung was analyzed along with the cellular components in the BALF and Th2 cytokine production in the BALF and splenocyte culture supernatant, lung histopathology, and goblet cell hyperplasia. The levels of OVA-specific IgE and IgG1, whose synthesis heavily depends on Th2 cells, were also measured in the serum. A single administration of α-GalCer in WT mice can increase the number of iNKT cells in the lung (Fig 1A and 1B) and also increases the production of IL-4 and IFN-γ in the lung iNKT cells (Fig 1C and 1D), compared with the PBS treatment (*P* < 0.05 or *P* < 0.01). Compared with the PBS treatment, α-GalCer administration did not result in lung infiltration of inflammatory cells, mucus production, and PAS-positive cells in the airways of WT mice (Fig 2A). No difference was observed in the BALF cell counts between mice treated with PBS and α-GalCer (Fig 2B) (*P* > 0.05). In addition, the levels of IL-4, IL-5, and IL-13 in the BALF and splenocyte culture supernatant were similar in mice treated with PBS and α-GalCer (Fig 2C and 2D) (*P* > 0.05). OVA-specific IgE and IgG1 in the serum were not detectable. Thus, α-GalCer administration can activate iNKT cells in WT mice, but the activation of iNKT cells alone cannot induce the Th2 inflammatory response without OVA immunization.

### iNKT cells are activated in the OVA-induced asthma model

**Fig 3:**
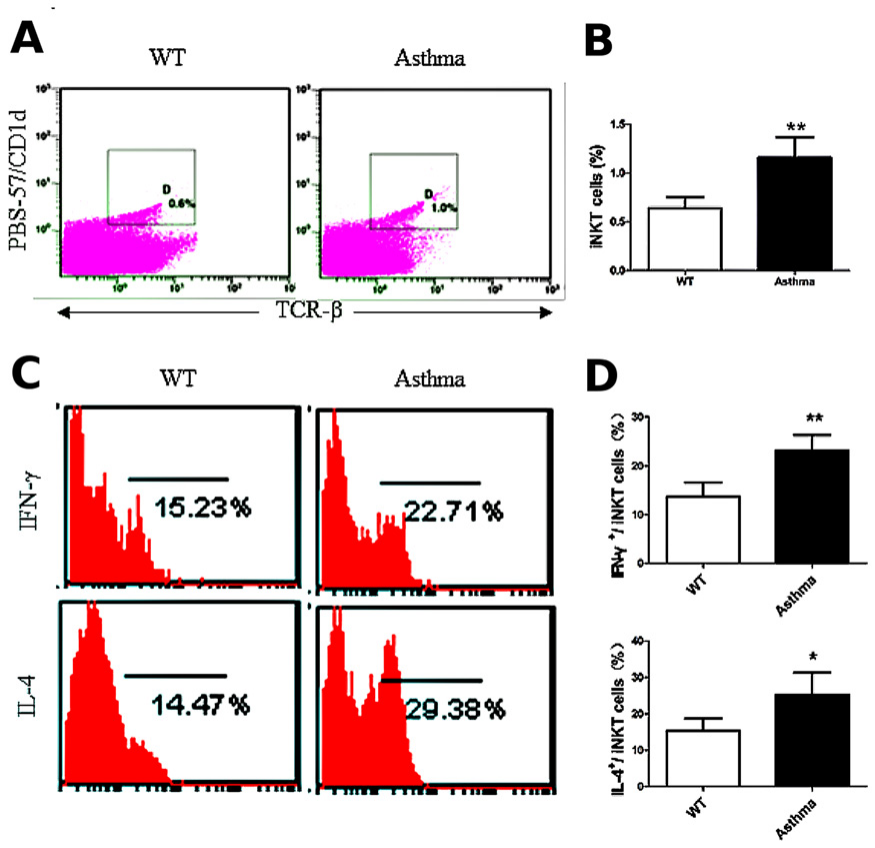
iNKT cells are activated in the OVA-induced asthma model. A. The percentage of iNKT cells (PBS-57/CD1d^+^ TCR-β^+^) in lung MNCs. The gating used for iNKT cells (gate D) and the corresponding percentages are shown in each dot plot. **B**. Percentage of iNKT cells in lung MNCs from OVA-induced asthmatic mice and WT mice. N = 5 per group and **P < 0.01. **C**. Lung iNKT cells producing IFN-γ and IL-4. PBS-57/CD1d and TCR-β double-positive cells were examined for IFN-γ (top) and IL-4 (bottom) secretion. Numbers in each dot plot indicate the percentage of positive cells. **D**. Percentage of IFN-γ- and IL-4-expressing lung iNKT cells. N = 5 per group. *P < 0.05, **P < 0.01.

OVA is a peptide antigen characterized by the absence of epitopes that activate iNKT cells. Nevertheless, we investigated whether iNKT cells were activated in the lungs of the OVA-induced asthma model. The percentage of iNKT cells in the lung MNCs increased (Fig 3A and 3B), and the production of IL-4 and IFN-γ in the lung iNKT cells also significantly increased (Fig 3C and 3D) in the OVA-induced asthmatic mice, compared with the WT mice (*P* < 0.05 or *P* < 0.01). Collectively, our data indicate that iNKT cells are activated in the OVA-induced asthma model.

### Activation of iNKT cells by α-GalCer enhances the Th2 inflammatory response in the OVA-induced asthma model

**Fig 4:**
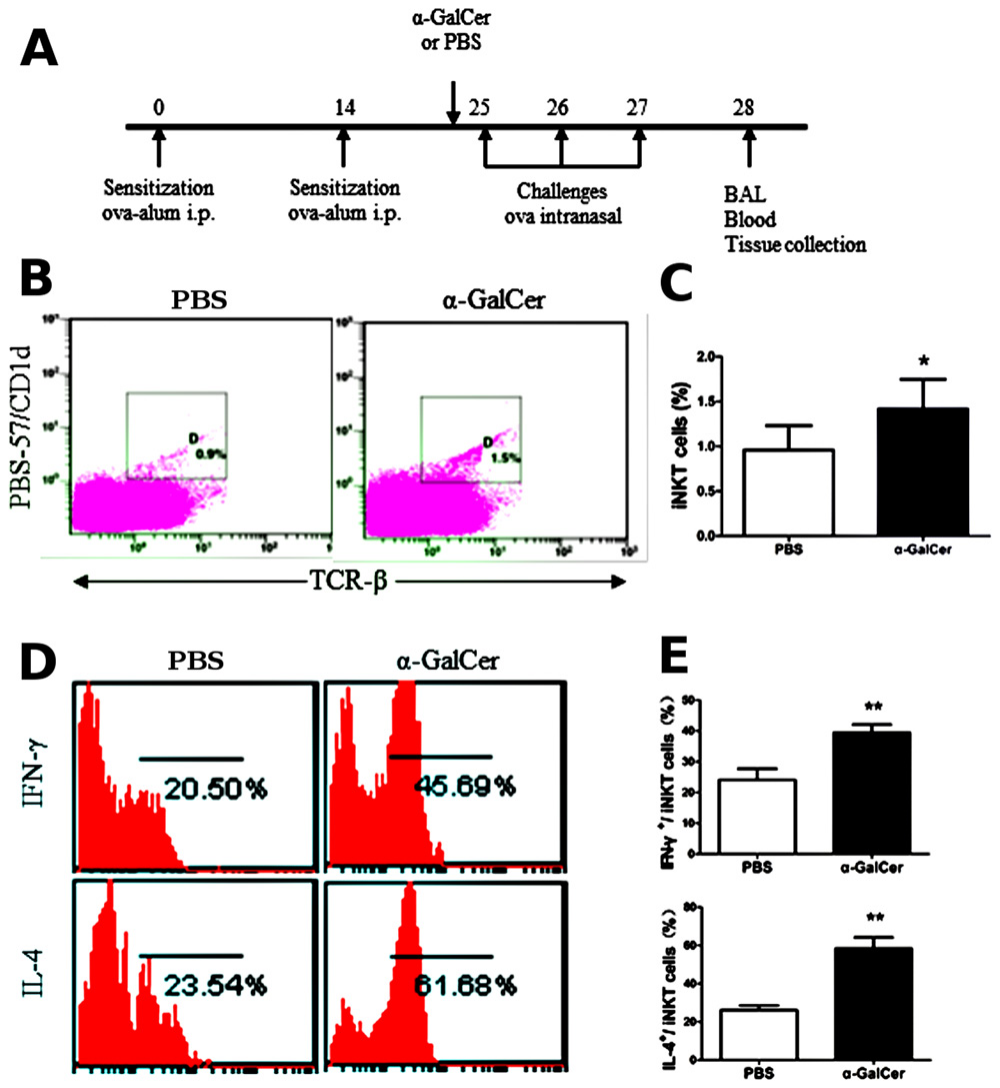
α-GalCer administration augments the activation of iNKT cells in OVA-induced asthmatic mice. **A**. Timeline of the OVA immunization/challenge protocol and the names of the treatment groups (α-GalCer or PBS). Mice were sacrificed 24 h after the final challenge and the number and activity of iNKT cells was analyzed in the lung. **B**. Lung iNKT cells were confirmed by PBS/CD1d tetramers and TCR-β staining in asthmatic mice treated with α-GalCer or PBS using flow cytometry. The gating used for iNKT cells (gate D) and the corresponding percentages are shown in each dot plot. **C**. Percentage of iNKT cells in lung MNCs from asthmatic mice treated with α-GalCer or PBS. N = 5 per group and *P < 0.05. **D**. Lung iNKT cells producing IFN-γ and IL-4 from asthmatic mice treated with α-GalCer or PBS. PBS-57/CD1d and TCR-β double-positive cells were examined for IFN-γ (top) and IL-4 (bottom) secretion. Numbers in each dot plots indicate the percentage of positive cells. **E**. Percentage of IFN-γ- and IL-4-expressing lung iNKT cells from asthmatic mice treated with α-GalCer or PBS. N = 5 per group and **P < 0.01.

**Fig 5:**
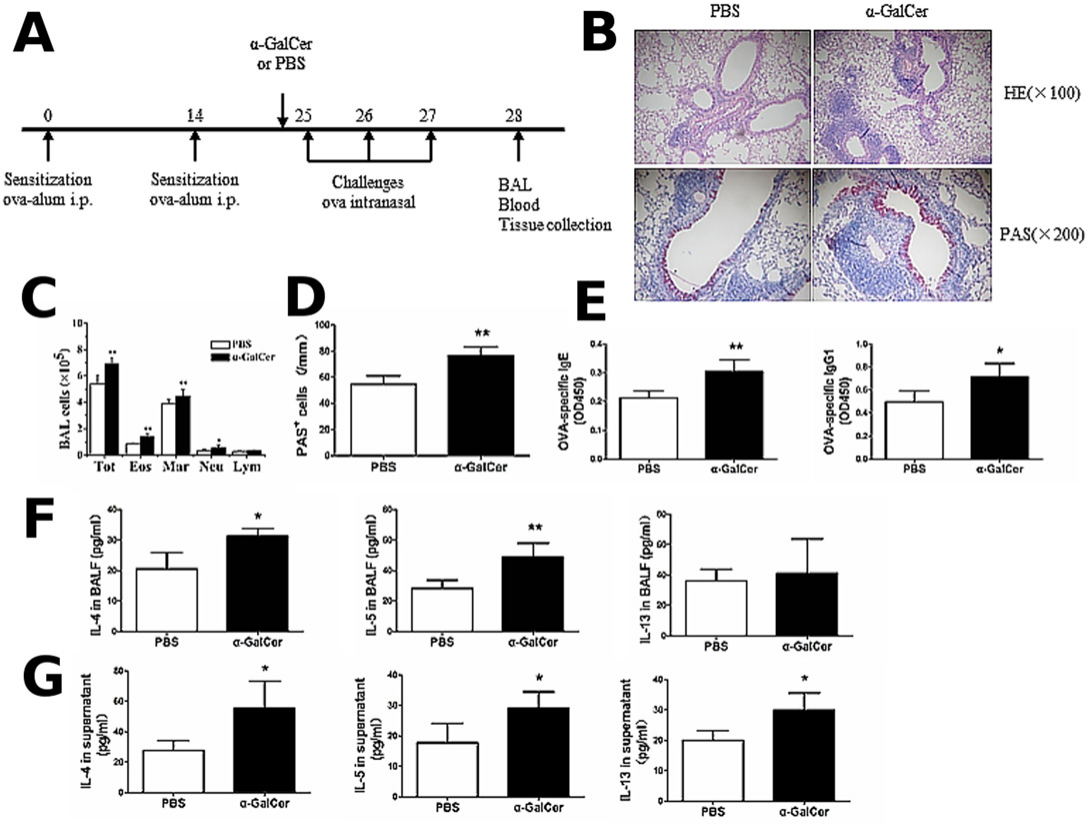
α-GalCer administration enhances the Th2 inflammatory response in OVA-induced asthma model. **A**. Timeline of the OVA immunization/challenge protocol and the names of the treatment groups (α-GalCer or PBS). **B**. Histopathological analysis using hematoxylin-eosin (H&E) staining and periodic acid-Schiff (PAS) staining of the lung tissue sections from OVA-induced asthmatic mice treated with α-GalCer or PBS. **C**. Total cell and differential cell counts in the BALF from OVA-induced asthmatic mice treated with α-GalCer or PBS were obtained by a standard hemocytometer. N = 4 per group and *P < 0.05, **P < 0.01. Tot, total cell counts; Mar, macrophages; Eos, eosinophils; Neu, neutrophils; and Lym, lymphocytes. **D**. Lung goblet cell hyperplasia is expressed as the number of PAS-positive cells per unit of length (mm) of the basement membrane. N = 4 per group and **P < 0.01. **E**. OVA-specific Ig levels from OVA-induced asthmatic mice treated with α-GalCer or PBS were analyzed by ELISA. N = 4–5 per group and *P < 0.05, **P < 0.01. **F**. BALF were collected and cytokine (IL-4, IL-5, and IL-13) production from OVA-induced asthmatic mice treated with α-GalCer or PBS was analyzed by ELISA. N = 4 per group and *P < 0.05, **P < 0.01. **G**. Splenocytes were obtained and re-stimulated with 500 μg OVA *in vitro* from OVA-induced asthmatic mice treated with α-GalCer or PBS. After 72 h, culture supernatants were collected and cytokine production was analyzed by ELISA. N = 4 per group and *P < 0.05.

To investigate the role of iNKT cells in Th2 inflammatory responses in the OVA-induced asthma model, we analyzed the Th2 inflammatory response in asthmatic mice that were intraperitoneally injected with 2 μg α-GalCer or PBS, 1 h before challenge on day 25. The mice were sacrificed 24 h after the final challenge and the number and activity of iNKT cells was analyzed in the lung. The OVA sensitization/challenge and α-GalCer treatment protocol are shown in the Fig 4A. As shown in Fig 4B and 4C, the percentage of iNKT cells was significantly increased in the lungs of OVA-induced asthma model treated with α-GalCer, compared with asthmatic mice treated with PBS (*P* < 0.05). Furthermore, α-GalCer administration increased the production of cytokines including IFN-γ and IL-4 (Fig 4D and 4E) in the lung iNKT cells of the OVA-induced asthmatic mice, compared with asthmatic mice treated with PBS (*P* < 0.01). Next, we analyzed the Th2 inflammatory response in asthmatic mice treated with α-GalCer and PBS. The OVA sensitization/challenge and α-GalCer treatment protocol are shown in the Fig 5A. Compared with asthmatic mice treated with PBS, there was marked inflammatory cell infiltration in the lung (Fig 5B) and a significant increase in the number of eosinophils, macrophages, neutrophils, and total cells in the BALF (Fig 5C) (*P* < 0.05 or *P* < 0.01). In addition, marked mucus production (Fig 5B) and PAS-positive goblet cells in the epithelium of the airway (Fig 5D) (*P* < 0.01), and increased serum levels of OVA-specific IgE and OVA-specific IgG1 were also observed in the α-GalCer-treated mice (Fig 5E) (*P* < 0.05 or *P* < 0.01). The levels of IL-4 and IL-5 in the BALF in asthmatic mice treated with α-GalCer were higher than in asthmatic mice treated with PBS (Fig 5F) (*P* < 0.05 or *P* < 0.01). OVA-stimulated splenocytes from asthmatic mice treated with α-GalCer showed increased production of IL-4, IL-5, and IL-13, as compared with cells from asthmatic mice treated with PBS (Fig 5G) (*P* < 0.05). Taken together, these results indicate that activation of iNKT cells by α-GalCer enhances the Th2 inflammatory response in asthma in the OVA-induced asthma mice.

### Adoptive transfer of iNKT cells enhances the Th2 inflammatory response in OVA-induced asthma model

**Fig 6:**
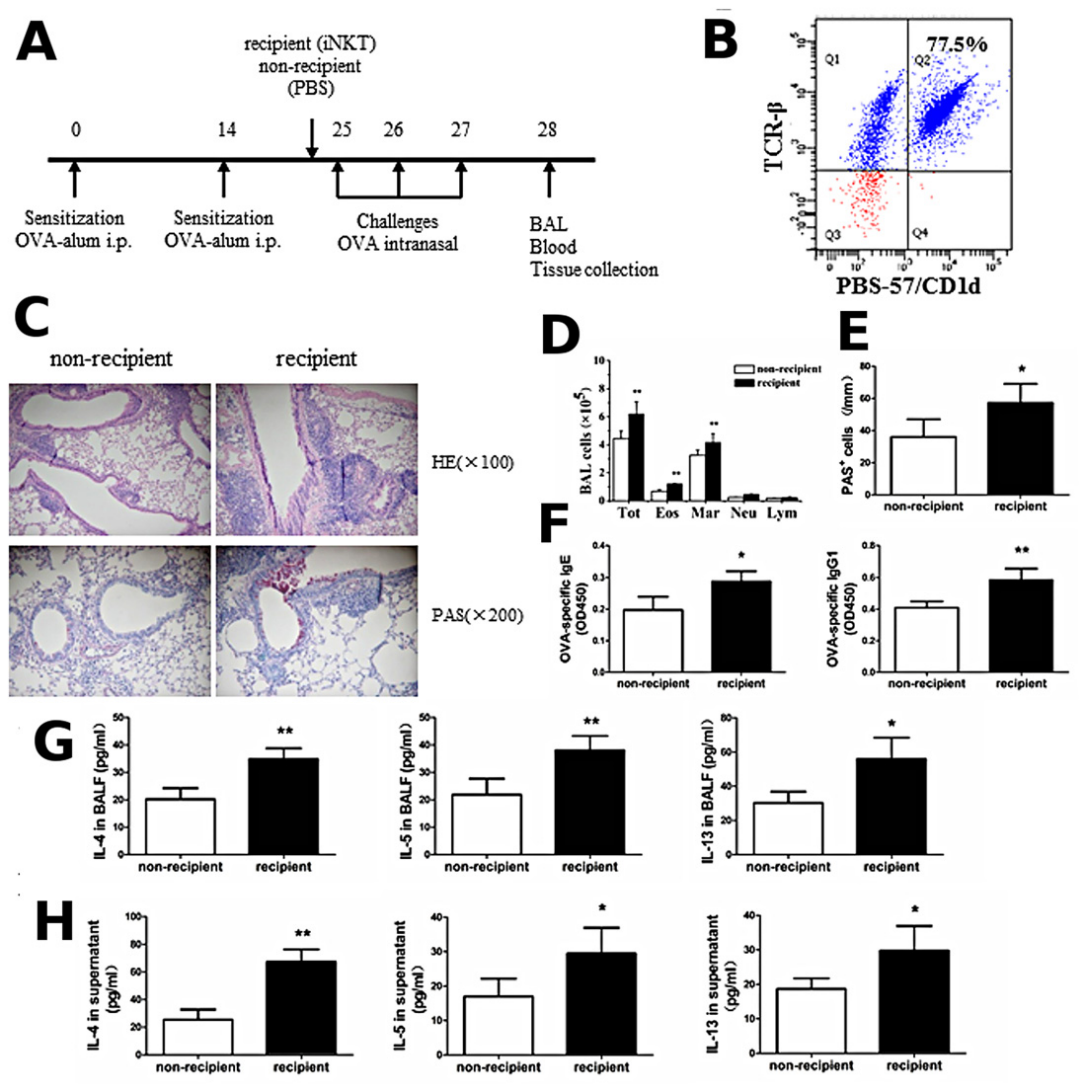
Adoptive transfer of iNKT cells enhances the Th2 inflammatory response in OVA-induced asthma model. **A**. Timeline of the OVA immunization/challenge protocol and the names of the treatment groups (PBS or iNKT cells). **B**. Purity of the adoptively transferred iNKT cell population. Flow cytometry shows 77.5% of the adoptively transferred cells stained with both PBS-57/CD1d tetramers and monoclonal antibody against TCR-β. **C**. Histopathological analysis using hematoxylin-eosin (H&E) staining and periodic acid-Schiff (PAS) staining of the lung tissue sections from OVA-induced asthmatic mice treated with adoptive transfer of iNKT cells or PBS. **D**. Total cell and differential cell counts in the BALF from OVA-induced asthmatic mice treated with adoptive transfer of iNKT cells or PBS were obtained by a standard hemocytometer. N = 4 per group and **P < 0.01. Tot, total cell counts; Mar, macrophages; Eos, eosinophils; Neu, neutrophils; and Lym, lymphocytes. **E**. Lung goblet cell hyperplasia is expressed as the number of PAS-positive cells per unit of length (mm) of the basement membrane. N = 4 per group and *P < 0.05. **F**. OVA-specific Ig levels from OVA-induced asthmatic mice treated with adoptive transfer of iNKT cells or PBS were analyzed by ELISA. N = 4 per group and *P < 0.05, **P < 0.01. **G**. BALF were collected 24 h after the final challenge and cytokine (IL-4, IL-5, and IL-13) production from OVA-induced asthmatic mice treated with adoptive transfer of iNKT cells or PBS was analyzed by ELISA. N = 4 per group and *P < 0.05, **P < 0.01. **H**. Splenocytes were obtained from OVA-induced asthmatic mice treated with adoptive transfer of iNKT cells or PBS and re-stimulated with 500 μg OVA *in vitro*. After 72 h, culture supernatants were collected and cytokine production was analyzed by ELISA. N = 4 per group and *P < 0.05, **P < 0.01.

To further confirm that iNKT cells specifically enhanced Th2 inflammatory response in the OVA-induced asthma model, we adoptively transferred iNKT cells from the OVA-induced asthmatic mice into BALB/c mice via tail vein injections, 1 h before the OVA challenge on day 25. Mice were sacrificed 24 h after the final challenge and the iNKT cells were purified from spleen MNCs using PE-PBS57/CD1d tetramers. The OVA sensitization/challenge and adoptive transfer of iNKT cells protocol are outlined in the Fig 6A. Adoptive transfer of a cell population enriched for cells that bound PBS-57-loaded CD1d tetramer and TCR-β, i.e., iNKT cells (>76% tetramer and TCR-β double-positive; Fig 6B), significantly enhanced Th2 inflammatory responses in the OVA-induced asthma model. Specifically, compared with PBS treatment, increased inflammatory cell infiltration in the lung (Fig 6C) and a significant increase in the number of eosinophils, macrophages, and total cells in the BALF (Fig 6D) (*P* < 0.01), increased mucus production (Fig 6C) and PAS-positive goblet cells in the epithelium of the airway (Fig 6E) (*P* < 0.05), as well as increased OVA-specific IgE and IgG1 levels in the serum (Fig 6F) (*P* < 0.05 or *P* < 0.01) were observed in the OVA-induced asthmatic mice treated with adoptive transfer of iNKT cells. Furthermore, the levels of IL-4, IL-5, and IL-13 in the BALF and splenocyte culture supernatant were significantly increased in the OVA-induced asthmatic mice treated with adoptive transfer of iNKT cells (Fig 6G and 6H), compared with PBS treatment (*P* < 0.05 or *P* < 0.01). Thus, adoptive transfer of iNKT cells enhances the Th2 inflammatory response in the OVA-induced asthma model.

### The Th2 inflammatory response is reduced but not completely abrogated in CD1d^-/-^ mice immunized and challenged with OVA

**Fig 7:**
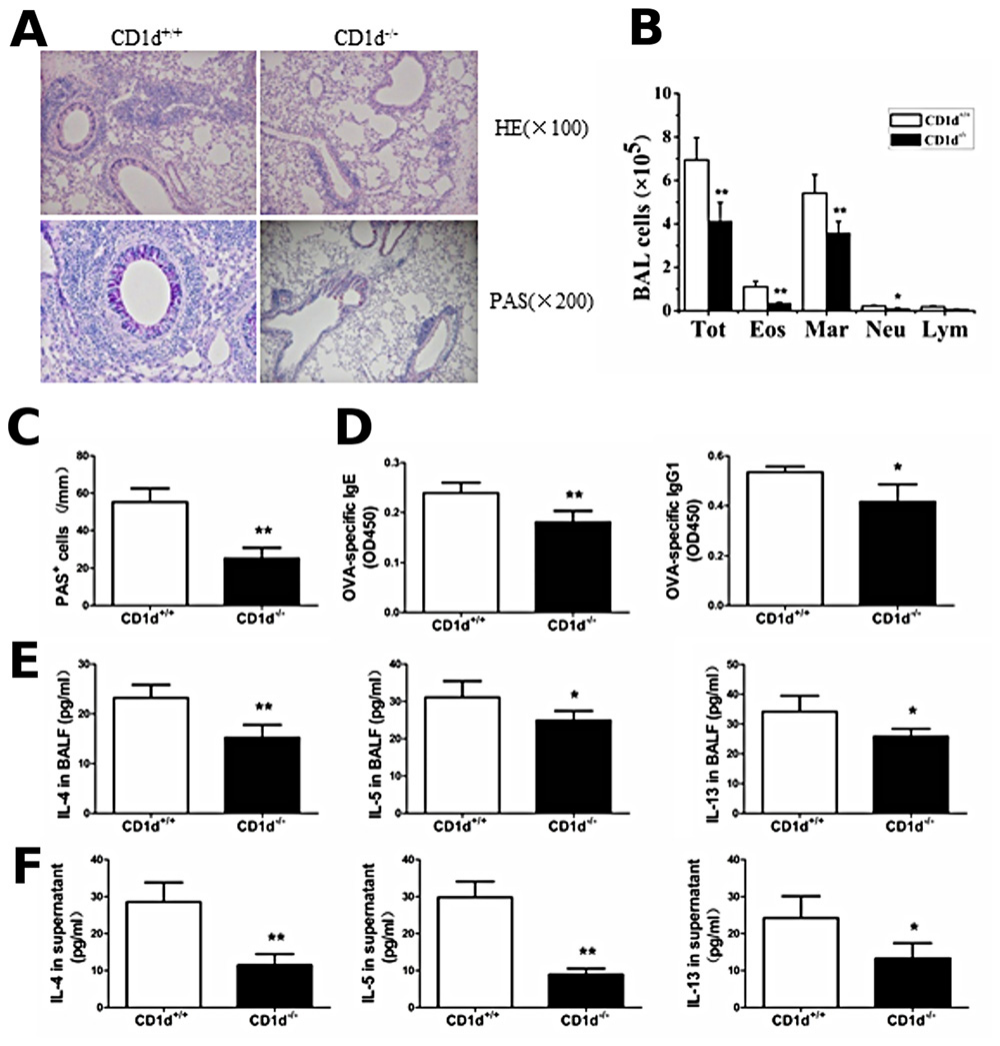
Th2 inflammatory response is reduced, but not completely abrogated in CD1d^-/-^ mice immunized and challenged with OVA, compared with WT (CD1d^+/+^) mice. **A**. Histopathological analysis using hematoxylin-eosin (H&E) staining and periodic acid-Schiff (PAS) staining of the lung tissue sections from CD1d^+/+^ and CD1d^-/-^ mice immunized and challenged with OVA. **B**. Total cell and differential cell counts in the BALF from CD1d^+/+^ and CD1d^-/-^ mice immunized and challenged with OVA were obtained by a standard hemocytometer. N = 4 per group and *P < 0.05, **P < 0.01. Tot, total cell counts; Mar, macrophages; Eos, eosinophils; Neu, neutrophils; and Lym, lymphocytes. **C**. Lung goblet cell hyperplasia is expressed as the number of PAS-positive cells per unit of length (mm) of the basement membrane. N = 4 per group and **P < 0.01. **D**. OVA-specific Ig levels from CD1d^+/+^ and CD1d^-/-^ mice immunized and challenged with OVA were analyzed by ELISA. N = 4 per group and *P < 0.05, **P < 0.01. **E**. BALF were collected 24 h after the final challenge and the levels of IL-4, IL-5, and IL-13 from CD1d^+/+^ and CD1d^-/-^ mice immunized and challenged with OVA were analyzed by ELISA. N = 4 per group and *P < 0.05, **P < 0.01. **F**. Splenocytes were obtained from CD1d^+/+^ and CD1d^-/-^ mice immunized and challenged with OVA and re-stimulated with 500 μg OVA *in vitro*. After 72 h, culture supernatants were collected and cytokine production was analyzed by ELISA. N = 4 per group and *P <0.05, **P < 0.01.

To determine the specific role of iNKT cells in the development of Th2 inflammatory responses in asthma, we examined the Th2 inflammatory response in CD1d^-/-^ mice, which lack the iNKT cells due to the absence of the class I restricting element of iNKT cells. As expected, CD1d^+/+^ mice (WT BALB/c mice) demonstrated marked airway inflammation when sensitized and challenged with OVA. In contrast, CD1d^-/-^ mice, when sensitized and challenged with OVA, showed decreased inflammatory cell infiltration in the lung (Fig 7A) and a significant decrease in the number of eosinophils, macrophages, neutrophils, and total cells in the BALF (Fig 7B) (*P* < 0.05 or *P* < 0.01), decreased mucus production (Fig 7A) and PAS-positive goblet cells in the airway epithelium (Fig 7C) (*P* < 0.01), as well as decreased serum levels of OVA-specific IgE and IgG1 (Fig 7D) (*P* < 0.05 or *P* < 0.01). In addition, the levels of IL-4, IL-5, and IL-13 in the BALF and splenocyte culture supernatant were significantly decreased in the CD1d^-/-^ mice (Fig 7E and 7F), compared with CD1d^+/+^ mice when sensitized and challenged with OVA (*P* < 0.05 or *P* < 0.01). Taken together, these data indicate that the Th2 inflammatory response is reduced, but not completely absent in CD1d^-/-^ mice immunized and challenged with OVA.

## Discussion

In the present study, we demonstrate that activation of iNKT cells alone cannot induce the Th2 inflammatory response, while α-GalCer administration can actually activate iNKT cells in WT mice without OVA immunization and challenge. However, activation of iNKT cells by α-GalCer and adoptive transfer of iNKT cells significantly enhances Th2 inflammatory responses in the OVA-induced asthma model. In addition, we show that the Th2 inflammatory response is reduced, but not completely abrogated in CD1d^-/-^ mice immunized and challenged with OVA, compared with WT mice. Our data indicate that iNKT cells are not essential, but may serve as an adjuvant to enhance the Th2 inflammatory response in the OVA-induced asthma model.

OVA is a peptide antigen characterized by the absence of epitopes that activate iNKT cells. Even so, this study shows that iNKT cells are activated in the OVA-induced asthma model, as evidenced by the increased percentage of iNKT cells and increased cytokine production. Consistent with our results, Akbari et al. [7] also reported that after OVA challenge, the percentage of iNKT cells in lung MNCs was increased and these iNKT cells produced a large amount of cytokines, including IL-4 and IL-13. The iNKT cells activation is achieved either through direct recognition of microorganism-derived exogenous lipid antigens or through indirect augmentation of the response of iNKT cells to endogenous lipid antigens under several specific conditions [18]. Several identified endogenous glycolipid or phospholipid antigens may stimulate subsets of type I semi-invariant iNKT cells, type II diverse NKT cells, or both [19–22]. During bacterial infection, iNKT cells are activated by the synergistic stimulation of self-lipids and proinflammatory cytokines [23]. Although this process has not been confirmed in asthmatic lung, it remains a possibility that iNKT cells could be activated by such a mechanism in asthma. Thus, the above results indicate that iNKT cells are involved in the development of the OVA-induced asthma model.

Multiple studies have implicated the role of iNKT cells in the development of asthma. However, whether iNKT cells are essential or just serve as an adjuvant in asthma remains controversial. In our study, α-GalCer administration activated iNKT cells, resulting in an increased percentage of iNKT cells and increased cytokine secretion in the lung. However, α-GalCer administration alone could not induce the Th2 inflammatory response in WT mice. Previous studies have demonstrated that, when challenged with antigens, CD1d^-/-^ and Jα18^-/-^ mice show reduced eosinophilic inflammation in the lung, compared with WT mice [7, 8], indicating that allergic airway inflammation can occur in mice deficient in iNKT cells. In addition, our previous study showed that allergic airway inflammation was reduced but not completely abrogated when the activity of iNKT cells was inhibited [15]. Furthermore, Wingender et al. [24] have reported that iNKT cells play a major role in mediating the Th2-biased adjuvant activities of house dust extracts in OVA-induced airway inflammation. Collectively, iNKT cells alone are not sufficient to induce the Th2 inflammatory response in asthma; thus, they are not essential but may play an adjuvant role in asthma.

We also investigated the effect of iNKT cells on Th2 inflammatory responses in the OVA-induced asthma model. The *in vivo* stimulation of NKT cells with α-GalCer significantly enhances the immune response against co-injected Ags [10–12]. Our results show that the activation of iNKT cells was augmented by α-GalCer in the OVA-induced asthma model, as indicated by the increased number of iNKT cells in the lung and elevated level of cytokine production. The Th2 inflammatory response was enhanced in the OVA-induced asthma model administrated with α-GalCer, compared with OVA-induced asthmatic mice with PBS administration. Furthermore, our study also shows that the adoptive transfer of iNKT cells augmented the Th2 inflammatory response in the OVA-induced asthma model. The above results indicate that the enhanced iNKT cell activity mediated by α-GalCer and the increased iNKT cell number can enhance the Th2 inflammatory response in OVA-induced asthmatic mice. To further clarify this role of iNKT cells, we employed CD1d^-/-^ mice to eliminate the activity of iNKT cells. CD1d^-/-^ mice showed remarkably reduced Th2 inflammatory response to OVA sensitization and challenge, as compared with WT mice. These results are consistent with a recent study which the OVA-specific effector CD4^+^ T cells that were adoptively transferred into naive CD1d^+/-^ mice exhibited stronger CD4^+^ Th1 and CD4^+^ Th2 immune responses to the OVA challenge, including increased proliferation and cytokine production, compared with the OVA-specific effector CD4^+^ T cells, when transferred into naive CD1d^-/-^ mice (NKT cell-deficient) [25]. The above results suggest that iNKT cells can enhance the Th2 inflammatory response in OVA-induced asthmatic mice.

The underlying mechanism of how iNKT cells enhance Th2 inflammatory responses in the OVA-induced asthma model remains unclear. However, a recent study has demonstrated the regulatory role of iNKT cells on DCs. The activation of iNKT cells by α-GalCer rapidly induces complete maturation of DCs and enhances CD4^+^ and CD8^+^ T cell response in mice [10]. In addition, Wiethe et al. [26] have reported that the state of differentiation of DCs and their interaction with NKT cells determines Th1/Th2 differentiation in the murine model of leishmania major infection. Shin et al. [25] found that the expression of the activation marker CD86 on DCs was higher in OVA-challenged CD1d^+/+^ mice, which were adoptive transferred with OVA-specific effector CD4^+^ T cells, than that in OVA-challenged CD1d^-/-^ recipient mice, suggesting that NKT cells boost the function of DCs *in vivo*. Similarly, our colleagues have found that α-GalCer administration increases the expression level of the maturation markers CD86 and MHC-II on DCs (data not shown). However, further studies are necessary to thoroughly understand how iNKT cells contribute to the *in vivo* Th2 inflammatory response by facilitating the functions of DCs.

In conclusion, our study demonstrates that iNKT cells are not essential, but may serve as an adjuvant to enhance the Th2 inflammatory response in an OVA-induced mouse model of asthma.
